# Exploring factors associated with pregnant women’s experiences of material hardship during COVID-19: a cross-sectional Qualtrics survey in the United States

**DOI:** 10.1186/s12884-021-04234-1

**Published:** 2021-11-08

**Authors:** Laura Johnson

**Affiliations:** School of Social Work, Temple University, Ritter Annex Room 543, 1301 Cecil B. Moore Avenue, Philadelphia, PA 19122 USA

**Keywords:** Pregnancy, COVID-19, Financial strain, Posttraumatic stress disorder, Intimate partner violence

## Abstract

**Background:**

The COVID-19 pandemic has exacerbated the financial insecurity of women and their families globally. Some studies have explored the impact of financial strain among pregnant women, in particular, during the pandemic. However, less is known about the factors associated with pregnant women’s experiences of material hardship.

**Methods:**

This cross-sectional study used a non-probability sample to examine the factors associated with pregnant women’s experiences of material hardship during the COVID-19 pandemic. In January 2021, 183 pregnant women living in the United States participated in an online Qualtrics panel survey. In addition to socio-demographic characteristics, individuals were asked about their finances and predictors of financial well-being, mental health symptoms, and intimate partner violence (IPV) experiences. Chi-square analysis and one-way ANOVA were used to examine whether women’s experiences with material hardship and associated factors differed by income level (i.e., less than $20,000; $20,000 to $60,000; more than $60,000). Ordinary least squares regression was used to calculate unadjusted and adjusted estimates.

**Results:**

Study findings showed that the majority of women in the sample experienced at least one form of material hardship in the past year. Individuals with an annual household income less than $20,000 reported the highest average number of material hardships experienced (*M* = 3.7, *SD* = 2.8). Compared to women with household incomes less than $20,000, women with incomes of more than $60,000 reported significantly fewer material hardships, less financial strain, and higher levels of financial support, economic self-efficacy, and economic-self-sufficiency. Women with incomes of $60,000 or more also reported significantly lower levels of psychological abuse, and a smaller percentage met the cut-off for anxiety. Economic self-sufficiency, financial strain, posttraumatic stress disorder, and economic abuse were all significantly associated with material hardship.

**Conclusions:**

A contribution of this study is that it highlights the significant, positive association between economic abuse, a unique form of IPV, and material hardship among pregnant women during the pandemic. These findings suggest the need for policy and practice interventions that help to ameliorate the financial insecurity experienced by some pregnant women, as well as respond to associated bidirectional vulnerabilities (e.g., mental health symptoms, experiences of IPV).

**Supplementary Information:**

The online version contains supplementary material available at 10.1186/s12884-021-04234-1.

## Background

In early 2020, the spread of severe acute respiratory syndrome coronavirus 2 (known as COVID-19) resulted in significant economic turmoil globally [1]. The United States experienced a stock market drop more significant than the Great Crash of 1929 [2] and in April 2020 unemployment rates peaked at 14.8% [3]. The pandemic has exacerbated labor market inequity, as individuals from racial and ethnic minority groups have been disproportionately impacted [3, 4], along with women [5].

Most studies exploring the impact of COVID-19 on women have focused on the general population [6]. However, pregnant women face additional stressors during natural disasters. During the COVID-19 pandemic, pregnant women have experienced increased uncertainty about the effects of the virus on maternal and perinatal health, and concerns about receiving medical treatment due to potential health risks [6, 7]. Women are also particularly vulnerable to increased financial stress and mental health symptomology [6]. Although several studies have documented the increased financial strain pregnant women have experienced as a result of COVID-19 [6–10], examining factors associated with this financial strain has not been their primary purpose. As such, the purpose of this study was to explore factors associated with pregnant women’s experiences of material hardship during the COVID-19 pandemic.

### Financial strain and pregnancy

Approximately 56% of people living in poverty in the United States are women and 31% are children [11]. Braveman and colleagues analyzed data from the Center for Disease Control’s Pregnancy Risk Assessment Monitoring System and found that over 50% of women in the sample were poor or near poor during their pregnancy based on the U.S. federal poverty level [12]. Further, 60% of these low-income women experienced at least one hardship (e.g., job loss; homelessness; intimate partner violence; food insecurity).

Pregnancy, itself, may cause increased financial stress, as mothers and families prepare for additional expenses related to childcare. Women may also experience financial losses during pregnancy as a result of employment changes [13]. Although some women choose to stop working, others are penalized by their employers for becoming pregnant or having a newborn; women are sometimes fired or lose out on opportunities for promotion as a result [14, 15]. During high-risk pregnancies, women may be prescribed bed rest prior to delivery, therefore further restricting their income generating activities and potentially causing additional household expenses (e.g., childcare costs) [16].

Although not often discussed, financial strain poses significant health risks to maternal and child health. For example, one study among pregnant women in South Africa found food insecurity was associated with depression, substance dependence, and anxiety [17]. Another study conducted in the United States found that financial strain was positively associated with depressive symptoms, anxiety, perceived stress, and pregnancy-specific distress, and negatively associated with birth weight; depression mediated the relationship between financial strain and birth weight [18].

Financial stress is also associated with intimate partner violence. Intimate partner violence refers to a pattern of coercive behaviors used by one intimate partner to control another [19]. Coercive tactics can be physical, sexual, psychological, or economic in nature. The relationship between financial insecurity and intimate partner violence is bidirectional; it serves as a risk factor for intimate partner violence perpetration [20] and for survivors it also serves as a primary barrier to leaving an abusive relationship [21, 22]. Although pregnancy functions as a protective factor for a sub-sample of women, for other survivors intimate partner violence may begin or persist during pregnancy [23]. The association between financial stress and intimate partner violence is particularly concerning, as intimate partner violence is also associated with a range of negative pregnancy-related outcomes including low birth weight, small for gestational age, perinatal death, and preterm birth [24–26].

### Increased stress during COVID-19 pandemic

The COVID-19 pandemic has exacerbated the financial stress of women and created unique challenges for pregnant women in particular. Overall, the pandemic has decreased the financial security of families. Since the COVID-19 pandemic began, over one-third of women have been laid off, furloughed, or received pay cuts [27]. Given that health insurance is often linked to employment in the United States, job loss may also result in lost health insurance coverage [9]. This may be particularly stressful for pregnant women, who have ongoing healthcare needs. Further, the financial insecurity caused by COVID-19 has forced some to utilize their savings, which now makes families more vulnerable to other unexpected financial hardships that may emerge [28].

The news media has highlighted ways in which the COVID-19 pandemic has made women’s experiences with economic abuse worse. Economic abuse is a form of intimate partner violence in which one intimate partner interferes with the other’s ability to access or acquire financial resources in order to increase their dependency [29]. In the United States, stimulus checks (approximately $600 per person) were provided to taxpayers making up to $150,000 for married couples and $75,000 for individuals; these stimulus checks were administered through bank direct deposit, by check, or in the form of a debit card [30]. However, these funds have been inaccessible to some women in abusive relationships. For example, the funds were directly deposited into their partners’ accounts, so women were unable to retrieve the money. As a result, women have lost access to a critical source of funding.

Women’s financial situations have been further strained by their emotional labor. Lock-down orders implemented to mitigate the risks of spreading COVID-19 have resulted in the closure of nurseries and schools, which has increased women’s caretaking responsibilities at home. Historically, these responsibilities have had a significant impact on women’s participation in the job market; women experience great lifetime wage penalties due to their caregiving responsibilities and may lose employment opportunities and promotions due to their emotional labor at home [31]. Given these and other barriers to financial security that women face as a result of pandemics, it takes women even longer to financially recover from outbreaks and other natural disasters than men [32, 33].

As a result of the significant stress women have experienced due to the COVID-19 pandemic, it is not surprising that researchers have reported a decline in mental health, including increased depression, anxiety, and posttraumatic stress disorder (see Almeida et al. for a review) [34]. This has also been found to be true of pregnant women, who are already considered high risk for mental disorders given their prevalence during the perinatal period [35]. Globally, several studies have looked at the impact of COVID-19 on the emotional well-being of pregnant women. Researchers found that pregnant women have had higher levels of depression [36–38], anxiety [36, 37], posttraumatic stress disorder [39] and increased thoughts of self-harm [38].

Scholars have also identified an association between pandemic-related financial strain and mental health concerns for pregnant women. Zhang & Ma found that pregnant women in their sample reported the impact of COVID-19 on their stress to be moderate to severe; stressors included finances, work, and increased responsibilities at home [6]. Thayer and Gildner reported that COVID-19 related financial stress was associated with increased likelihood of depression, even after controlling for income [9]. Preis et al. found that 45.8% of pregnant women in their sample lost income due to the pandemic and this income loss was associated with both pregnancy preparedness stress and perinatal infection stress [8].

Rates of intimate partner violence have also increased during the COVID-19 pandemic [40–42]. Naghizadeh and colleagues found one-third of their sample of 250 pregnant women seeking services at an obstetrics clinic in Iran reported experiencing intimate partner violence and those who had experienced abuse reported lower mental health quality of life [43]. Similarly, a study of 885 pregnant women in South Africa found approximately 12% of women fit the criteria to be classified as having probable common mental health disorders; a higher percentage of these women reported anxiety about being infected with COVID, were severely food insecure during the lockdown, and had experienced either psychological, physical, or sexual abuse by an intimate partner [44].

In summary, although financial stress is not uncommon among pregnant women, the COVID-19 pandemic has greatly exacerbated financial insecurity among women and their families. While some studies have explored the impact of financial strain among pregnant women during the COVID-19 pandemic, fewer have looked specifically at the factors most associated with women’s experiences with material hardship. As such, this study sought to answer three primary research questions: (1) To what extent did pregnant women in this sample experience material hardship during the past 1 year? (2) Did women’s experiences with material hardship and associated factors differ by income level? (3) What factors were most associated with women’s experiences with material hardship in the past 1 year? This study was exploratory in nature and sought to describe participants’ experiences.

## Methods

The data used in this analysis came from a cross-sectional study that examined perceptions of social support and financial well-being among a non-probability sample of pregnant women in the United States. Although not specifically a survey about the COVID-19 pandemic, a few questions on the financial impact of the pandemic were included to assess women’s service needs. Further, because most questions asked participants about their experiences in the past year, this time frame coincided with the emergence of the pandemic.

This study comes from a larger dataset, collected in January 2021 using Qualtrics research panel service. As part of Qualtrics panel service, the researcher worked with a program coordinator at Qualtrics to administer a survey to a pre-arranged and targeted pool of female-identified respondents. Recruitment was facilitated through Qualtrics via email and the survey was available exclusively to Qualtrics panel participants. Individuals interested participating were directed to a web-based survey which was built using the Qualtrics survey platform.

To be eligible to participate in the study, individuals needed to be: (a) age 18 years or older, (b) in a relationship, and (c) currently pregnant. Because the study was focused on financial well-being, quotas were designated so that approximately one-third of the sample would have an annual household income of less than $20,000, one-third would be between $20,000 and $60,000, and the remainder would have an income of more than $60,000. Participant eligibility was confirmed through a series of screening questions at the beginning of the survey.

All research procedures were approved by the Temple University Institutional Review Board. The survey began with informed consent, which outlined the anticipated length of time it would take to complete the survey (30 min), as well as the principal investigator and the purpose of the study. The informed consent also indicated that participation was voluntary and that responses were anonymous. Documentation of consent was waived to ensure participant anonymity. On average, the survey took approximately 20 min for participants to complete and they were compensated approximately $10, although the exact compensation rate was designated by Qualtrics.

Because some of the research questions asked participants about their romantic relationships, including intimate partner violence, a number of strategies were used to ensure participant safety. First, the survey was marketed as a study focused on social support and financial well-being among pregnant women. A range of resources were presented both at the beginning and end of the survey, including maternal health resources and hotlines for suicide prevention, sexual violence, and intimate partner violence. The informed consent also emphasized that internet usage can be monitored and impossible to erase completely, so if participants had concerns about their safety or privacy they could decline participation. There was also an emergency exit button at the bottom of each page, which would redirect the participant to a blank Google page if clicked on. As an added safety precaution, no back button was included on the survey.

The survey was developed using a series of validated measures. Adaptive questioning was used to reduce the number of items and complexity of items where appropriate. Most questions on the survey had binary or Likert scale response options. Overall, the survey questions were distributed across 20 pages. With the inclusion of conditional display, the minimum number items a survey participant would receive was 173 and the maximum was 190. The survey was piloted with 10% of total respondents prior to fielding. After the pilot, the Qualtrics program coordinator added a speeding check (measured as one-half the median soft launch time) which automatically terminated individuals who were not responding thoughtfully. The cut-off time was set at 6 min. To prevent the submission of multiple entries by the same respondent, Qualtrics placed a cookie on the participants’ browser when they submitted a survey response. Therefore, the next time that individual clicked on the survey link, Qualtrics could see this cookie and restrict them from taking the survey again. IP addresses were not used to track unique respondents to ensure the anonymity of participants. The completed Checklist for Reporting Results in Internet E-Surveys (CHERRIES) [45] can be found under Supplementary Materials.

### Measures

The descriptive characteristics of the measures used in this study are presented in Table 1.

**Table 1:** Characteristics of key measures

Variables	*M* (*SD*)	*F* (%)	Min.	Max.	*n of items*	α	Skewness	Kurtosis
Material hardship	2.5 (2.5)		0	11	11	–	1.1	.6
Financial support	4.0 (2.0)		0	6	6	–	−.6	−1.0
Economic self-efficacy	3.5 (.8)		1	5	10	.92	−.4	.1
Economic self-sufficiency	3.4 (.9)		1	5	8	.89	−.5	−.6
Financial strain	2.7 (1.0)		1	5	7	.90	.2	−.7
Anxiety, % yes		71 (38.8)	0	1	2	–	.5	−1.8
Depression, % yes		57 (31.2)	0	1	2	–	.8	−1.3
PTSD, % yes		70 (38.3)	0	1	5	–	.5	−1.8
Economic abuse	1.8 (1.0)		1	5	14	.97	1.1	−.1
Psychological abuse	2.0 (1.0)		1	5	10	.95	.9	−.3
Sexual abuse, % yes		20 (10.9)	0	1	1	–	2.5	4.4
Physical abuse, % yes		29 (15.9)	0	1	1	–	1.9	1.6

*M* Mean, *SD* Standard deviation, *F* Frequency, *PTSD* Posttraumatic stress disorder

#### Material hardship

The dependent variable used in this study was material hardship. Material hardship was measured using an index of 11 items that ask participants about their ability to meet basic financial needs over the past 12 months by indicating 1 (*yes*) or 0 (*no*)*.* Participants’ responses were then summed. The items were adapted from the Fragile Families study [46]. Example items include “In the past year did you get evicted for not paying rent/mortgage?” and “In the past year did you move in with people because of financial problems?”

#### Financial support

To measure perceptions of financial support, participants were asked if they could count on someone during the next year to provide various types of instrumental support to them by indicating 1 (*yes*) or 0 (*no*) [47]. The six items were: (a) loan you $200; (b) loan you $1000; (c) provide you with a place to live; (d) help with emergency child care; (e) cosign a $1000 loan; and (f) cosign a $5000 loan. Participants’ responses were them summed.

#### Economic self-efficacy

Economic self-efficacy was measured using the ten-item Scale of Economic Self-Efficacy [48]. Participants were asked to indicate the extent to which they agreed with a series of financial statements by indicating 1 (*strongly disagree*) to 5 (*strongly agree*). Example items include “I can always manage to solve difficult financial problems if I try hard enough” and “I am confident that I could deal efficiently with unexpected financial events.” Among this sample, this scale showed excellent reliability (α = .92).

#### Economic self-sufficiency

To measure economic self-sufficiency, the Scale of Economic Self-Sufficiency was used [49]. Participants were asked how often they were able to do a range of financial activities in the past 1 month (30 days) by indicating 1 (*not at all*) to 5 (*all the time*). Example items include “Pursue your own interests and goals” and “Meet your financial obligations.” This scale showed good reliability (α = .89).

#### Financial strain

Financial strain was measured using eight items from the 18-item Financial Strain Survey – Revised [49]. Specifically, four items came from the Physical Symptoms subscale and four items came from the Relationship Problems subscale. Participants were asked to indicate how often a series of items applied to them over the past 1 month (30 days). Response options ranged from 1 (*never*) to 5 (*always*). Example items include “Do your muscle get tense when you add up your bills?” and “Financial problems hurt my romantic relationships.” An exploratory factor analysis was conducted with this sample and the number of items was reduced from 8 to 7 (α = .90).

#### Depression and anxiety

Depression and anxiety were measured using the Patient Health Questionnaire-4 (PHQ-4) [50]. As part of the PHQ-4, participants were asked to indicate how often they had been bothered by a series of depressive and anxiety symptoms over the past 2 weeks. Response options ranged from 0 (*not at all*) to 3 (*nearly every day*). There are four items total; two measure depressive symptoms (i.e., little interest or pleasure in doing things; feeling down, depressed, or hopeless) and two measure anxiety symptoms (i.e., feeling nervous, anxious, or on edge; not being able to stop or control worrying). Individuals were coded as showing symptoms of depression or anxiety (binary *yes* or *no*) if they had a score equal to or greater than three on that respective subscale.

#### Posttraumatic stress disorder

Posttraumatic stress disorder was measured using the Primary Care PTSD screen (PC-PTSD) [51]. Participants were asked to indicate whether they had experienced the following symptoms by indicating 1 (*yes*) or (*no*): (a) had nightmares about the event(s) or thought about the event(s) when you did not want to; (b) tried hard not to think about the event(s) or went out of your way to avoid situations that reminded you of the event(s); (c) been constantly on guard, watchful, or easily startled; (d) felt numb or detached from people, activities, or your surroundings; and (e) felt guilty or unable to stop blaming yourself or others for the event(s) or any problems the event(s) may have caused? Participants’ score was summed and were coded as showing symptoms of posttraumatic stress disorder (binary *yes* or *no*) if they had a score equal to or greater than four, which is considered to be a more conservative cutoff.

#### Economic abuse

Economic abuse was measured using the 14-item Revised Scale of Economic Abuse (SEA2) [29]. Participants were presented with a range of behaviors that individuals sometimes use to control their partners financially and were asked to select the answer that best represents their personal experience in the past 12 months by indicating how often each behavior occurred. The response options ranged from 1 (*never*) to 5 (*quite often*). Items included “Decide how you could spend money rather than letting you spend it how you saw fit” and “Force or pressure you to give them savings or other assets.” The SEA-2 is comprised of two factors, economic restriction and economic exploitation; however, the full scale was used for this analysis (α = .97).

#### Psychological abuse

Psychological abuse was measured using the Psychological Abuse subscale from the Abusive Behavior Inventory-R2 (ABI-R2) [49]. Participants were presented with a range of behaviors that individuals sometimes use to control their partners and were asked to select the answer that best represents their experience in the past 12 months by indicating how often each behavior occurred. The response options ranged from 1 (*never*) to 5 (*quite often*). Items included “Told you that you were a bad person” and “Said things to scare you.” Overall, the ten items demonstrated excellent reliability (α = .95).

#### Physical and sexual abuse

Physical and sexual abuse were measured using questions adapted from the Two-Question Screening Tool [52]. This screening tool was developed for nurses in medical settings. The original questions were “Have you ever been hit, slapped, kicked, or otherwise physically hurt by your male partner?” and “Have you ever been forced to have sexual activities?” These questions were adapted to make them gender neutral and asked participants about their experiences within the past 12 months. Response options for these questions were 1 (*yes*) or 0 (*no*). Each item was examined individually.

#### Socio-demographic characteristics

Demographic characteristics included in this analysis were participants’ age (in years), sexual orientation (whether participant was in an opposite-sex relationship), race (White, Black or African American, Another), Latinx (*yes* or *no*) and education level (high school degree or lower, some college, bachelor’s degree or higher). Participants were also asked whether they were currently employed (*yes* or *no*), income level (less than $20,000, $20,000 to $60,000, or more than $60,000), number of children, and whether their employment status or the employment status of their partner changed as a result of the COVID pandemic (*yes* or *no*).

### Analysis plan

The analyses began with a missing value analysis in SPSS. This analysis showed that missing values were less than 1.5% across all variables. Therefore, participants who had missing values on the variables of interest in this study were removed from the sample. This reduced the sample size from 210 to 183. Descriptive statistics are presented for all measures included in regression model. In addition to examining the characteristics of the full sample, descriptive statistics were also calculated based on participants’ income at three levels: Less than $20,000, $20,000 to $60,000 and $60,000 or more. Pearson’s chi-square tests and one-way analysis of variance (ANOVA) were used to look at differences across income groups on demographic characteristics, material hardship, and associated factors. A Bonferroni adjustment was made to correct for multiple pairwise comparisons (2 or more). Ordinary least squares (OLS) regression was used to calculate unadjusted and adjusted estimates. These analyses were run in StataMP 16. The unadjusted model examined bivariate associations between material hardship and variables of interest (i.e., socio-demographic characteristics, financial predictors, mental health symptoms, abuse experiences) whereas the adjusted model included covariates. Post-hoc power analysis showed that the sample size was adequate for detecting an effect size of .39 (medium) with power set to .8 and a significance level of .05 (two-tailed) for the number of predictors (21 predictors) in the adjusted model.

## Results

Participants’ demographic characteristics are presented in Table 2. Across the full sample, the average age of participants was 28 years (*SD* = 5.9). The majority of participants identified as white (77%); 23% identified as Latinx. Most participants identified as being in an opposite-sex relationship (83%). With regard to education, 30% had a high school degree or lower, 28% completed some college, and 42% had earned a bachelor’s degree or higher. Consistent with the annual household income quotas, 28% of participants had an annual household income of less than $20,000, 36% were between $20,000 and $60,000 and 36% had an annual household income of more than $60,000. On average, participants had one child (*SD* = 1.1). A little over half the sample (64%) were employed either full or part-time; however, 46% of participants reported that their employment status had changed as a result of the COVID-19 pandemic. Similarly, participants reported that 41% of their partners also had their employment status change as a result of the pandemic Participants differed significantly by income level on age, whether they were in an opposite-sex relationship, education level, whether they were employed, and whether the participant or their romantic partner had a change in employment status as a result of the COVID-19 pandemic.

**Table 2:** Descriptive characteristics of participants by income (*n* = 183)

Variable	Full sample (*n* = 183)		Income Level						Group differences *p* value
			Less than $20,000 (*n* = 52)		$20,000 - $60,000 (*n* = 65)		More than $60,000 (*n* = 66)		
	*M* (SD)	*F* (%)	*M* (SD)	*F* (%)	*M* (SD)	*F* (%)	*M* (SD)	*F* (%)	
Age	28.3 (5.9)		25.0 (5.8)		27.6 (5.3)		31.6 (4.8)		*p* ≤ .017
Race									NS
Black/African American		18 (9.8)		6 (11.5)		7 (10.8)		5 (7.6)	
White		141 (77.0)		38 (73.1)		52 (80.0)		51 (77.3)	
Another		24 (13.1)		8 (15.4)		6 (9.2)		10 (15.2)	
Latinx, % yes		42 (23.0)		10 (19.2)		21 (32.3)		11 (16.7)	NS
Opposite-sex relationship, % yes		151 (82.5)		33 (63.5)		54 (83.1)		64 (97.0)	*p* ≤ .017
Education level, % yes									*p* ≤ .006
H.S. degree or lower		55 (30.1)		33 (63.5)		21 (32.3)		1 (1.5)	
Some college		52 (28.4)		18 (34.6)		24 (36.9)		10 (15.2)	
Bachelor’s degree or higher		76 (41.5)		1 (1.9)		20 (30.8)		55 (83.3)	
Employed, % yes		117 (63.9)		17 (32.7)		29 (44.6)		60 (90.9)	*p* ≤ .008
Number of children	1.0 (1.1)		.9 (1.0)		1.1 (1.4)		.98 (1.0)		NS
Employment status changed due to the COVID pandemic, % yes		84 (45.9)		31 (59.6)		33(50.8)		20 (30.3)	*p* ≤ .008
Partner’s employment status changed due to the COVID pandemic, % yes		75 (41.0)		31 (59.6)		29 (44.6)		15 (22.7)	*p* ≤ .008

Bonferroni adjustment was used to correct for multiple comparisons (2 or more)

*NS* Not statistically significant

Descriptive information about participants’ experiences with material hardship is presented in Table 3. Overall, 73% of the sample experienced at least one form of material hardship in the past 12 months. On average, participants experienced approximately two forms of hardship (*M* = 2.5; *SD* = 2.5). The most frequently experienced form of material hardship was receiving free food or meals (41%), followed by borrowing money from family or friends to pay bills (36%), and not paying the full amount of rent or mortgage (30%). The least frequently experienced form of material hardship was getting evicted for not paying rent or mortgage, which was experienced by approximately 9% of the sample.

**Table 3:** Experiences of material hardship in the past 12 months by frequency and percentage (*n* = 183)

Variable	Full Sample (*n* = 183)	Income			Group differences *p* value
		Less than $20,000 (*n* = 52)	$20,000 - $60,000 (*n* = 65)	More than $60,000 (*n* = 66)	
Did you receive free food or meals?	69 (40.6)	25 (48.1)	20 (30.8)	24 (36.4)	NS
Did you borrow money from family or friends to pay bills?	63 (36.0)	29 (55.8)	27 (41.5)	7 (10.6)	*p* ≤ .008
Did you not pay the full amount of rent/mortgage?	50 (30.3)	21 (40.4)	15 (23.1)	14 (21.2)	*p* ≤ .008
Did you not pay full/gas/oil/electric bill?	49 (28.5)	16 (30.8)	18 (27.7)	15 (22.7)	NS
Did you go hungry?	44 (24.9)	19 (36.5)	14 (21.5)	11 (16.7)	NS
Did anyone in the house need medication but couldn’t get them because of cost?	37 (21.4)	19 (36.5)	11 (16.9)	7 (10.6)	*p* ≤ .008
Move in with people because of financial problems?	36 (21.1)	17 (32.7)	11 (16.9)	8 (12.1)	*p* ≤ .008
Did your telephone service get disconnected for nonpayment?	34 (19.5)	17 (32.7)	12 (18.5)	5 (7.6)	*p* ≤ .008
Stay in a shelter, abandoned building, or car for even one night?	28 (16.2)	13 (25.0)	7 (10.8)	7 (10.6)	NS
Did your gas/electric/oil get shut off or withheld?	23 (13.5)	10 (19.2)	9 (13.8)	4 (6.1)	NS
Did you get evicted for not paying rent/mortgage?	15 (9.2)	6 (11.5)	4 (6.2)	5 (7.6)	NS
Average number of material hardships experienced	2.5 (2.5)	3.7 (2.8)	2.3 (2.2)	1.6 (2.2)	*p* ≤ .008

Bonferroni adjustment was used to correct for multiple comparisons (2 or more)

*NS* Not statistically significant

When material hardship was examined by income level, there were statistically significant differences by income level on 5 of the 11 material hardship items. Across all of these items, individuals at the income level of less than $20,000 had the highest percentage of participants respond affirmatively to: (a) borrowing money from family or friends to pay bills (56%), (b) not paying the full amount of rent/mortgage (40%), (c) being unable to get medication someone in the household needed because of cost (37%), (d) moving in with people because of financial problems (33%), and (e) having telephone service disconnected for nonpayment (33%). In addition, individuals at this income level reported the highest average number of material hardships experienced (*M* = 3.7, *SD* = 2.8).

Prior to conducting the OLS regression, factors associated with material hardship were also examined by income level. These findings are presented in Table 4. Overall, there were statistically significant differences across groups on most of the key variables of interest.

**Table 4:** Descriptive characteristics on key measures by income (*n* = 183)

Variable	Income Level						Group differences *p* value
	Less than $20,000 (*n* = 52)		$20,000 - $60,000 (*n* = 65)		More than $60,000 (*n* = 66)		
	*M* (SD)	*F* (%)	*M* (SD)	*F* (%)	*M* (SD)	*F* (%)	
Material hardship	3.7 (2.8)		2.3 (2.2)		1.6 (2.2)		*p* ≤ .017
Financial support	3.1 (2.1)		3.7 (1.9)		5.1 (1.6)		*p* ≤ .017
Economic self-efficacy	3.2 (.9)		3.4 (.7)		4.0 (.7)		*p* ≤ .017
Economic self-sufficiency	2.8 (.9)		3.3 (.8)		3.8 (.8)		*p* ≤ .017
Financial strain	3.1 (1.0)		2.8 (1.0)		2.3 (.9)		*p* ≤ .017
Anxiety, % yes		28 (53.8)		26 (40.0)		17 (25.8)	*p* ≤ .008
Depression, % yes		22 (42.3)		19 (29.2)		16 (24.2)	NS
PTSD, % yes		28 (53.8)		24 (36.9)		18 (27.3)	NS
Economic abuse	2.0 (1.1)		1.7 (1.0)		1.6 (1.0)		NS
Psychological abuse	2.4 (1.1)		2.0 (1.1)		1.6 (.8)		*p* ≤ .017
Sexual abuse, % yes		5 (9.6)		8 (12.3)		7 (10.6)	NS
Physical abuse, % yes		9 (17.3)		13 (20.0)		7 (10.6)	NS

Bonferroni adjustment was used to correct for multiple comparisons (2 or more)

*M* Mean, *SD* Standard deviation, *PTSD* Post-traumatic stress disorder, *NS* Not statistically significant

Compared with women with household incomes less than $20,000, women with incomes of more than $60,000 reported significantly fewer material hardships and lower levels of financial strain, and higher levels of financial support, economic self-efficacy, and economic-self-sufficiency. Women with household incomes more than $60,000 also reported significantly lower levels of psychological abuse than women with households of less than $20,000. Lastly, a smaller percentage (26%) of women with household incomes more than $60,000 met the cutoff for anxiety; more than half (54%) of women with annual household incomes less than $20,000 met the cutoff for anxiety.

In the unadjusted model, a range of financial well-being indicators, mental health, and intimate partner violence experiences were significantly associated with material hardship in the past 12 months (see Model 1 in Table 5). Being in an opposite-sex relationship, education (bachelor’s degree or higher compared to high school degree or lower), income level ($20,000 to $6000 or more than $60,000 compared to less than $20,000), having financial support, higher levels of economic self-efficacy and higher levels of economic self-sufficiency were all associated with decreased (fewer) material hardships, whereas change in employment status or change in partners’ employment status during COVID-19 pandemic, financial strain, anxiety, depression, posttraumatic stress disorder, and all four forms of intimate partner violence (i.e., economic, psychological, physical, and sexual) were associated with increased (more) material hardships in the unadjusted models.

**Table 5:** Ordinary least squares regression predicting material hardship (*n* = 183)

Variable	Model 1 Unadjusted			Model 2 Adjusted		
	*B*	95% CI		*B*	95% CI	
		LL	UL		LL	UL
*Demographic Characteristics*						
Age	−.05	−.11	.01	.03	−.02	.09
Race (ref: White)						
Black or African American	1.03	−.21	2.26	.06	−.94	1.05
Another	−.11	−1.20	.98	−.05	−.89	.79
Latinx	.47	−.40	1.34	.04	−.65	.74
Education (ref: HS degree or lower)						
Some college	−.70	−1.61	.20	−.09	−.90	.72
Bachelor’s degree or higher	− 1.94***	−2.77	− 1.11	−.47	− 1.44	.49
Opposite-sex relationship	− 1.27**	−2.22	−.33	−.42	− 1.23	.39
Number of children	.27	−.05	.59	−.09	−.36	.17
*Financial Well-Being*						
Employed	−.67	− 1.43	.08	−.26	−.99	.48
Income level						
$20,000 - $60,000	−1.42**	−2.29	−.54	−.46	− 1.29	.37
More than $60,000	−2.06***	−2.93	−1.19	−.03	−1.20	1.13
Employment status changed during COVID pandemic	1.44***	.73	2.14	.04	−.60	.67
Partner’s employment status changed during COVID pandemic	1.88***	1.19	2.58	.64	.00	1.27
Financial support	−.48***	−.65	−.32	−.08	−.26	.10
Economic self-efficacy	−1.02***	−1.44	−.60	.16	−.30	.63
Economic self-sufficiency	−1.18***	−1.54	−.83	−.61**	− 1.05	−.16
Financial strain	1.30***	1.00	1.61	.43*	.03	.83
*Mental Health*						
Anxiety	1.45***	.73	2.18	−.31	−1.06	.43
Depression	1.49***	.73	2.25	−.05	−.82	.71
Posttraumatic stress disorder	2.44***	1.78	3.11	1.06**	.34	1.77
*Intimate Partner Violence Experiences*						
Economic abuse	1.30***	.99	1.60	1.01***	.51	1.51
Psychological abuse	1.19***	.88	1.50	−.38	−.88	.13
Physical abuse	2.95***	2.04	3.86	.54	−.45	1.53
Sexual abuse	1.97**	.83	3.11	.10	−.94	1.13
Constant				1.45	−1.22	4.11
*R*^2^				.54		

Adjusted model controls for all other variables in the regression model

*CI* Confidence interval, *UL* Upper limit confidence interval, *LL* Lower limit confidence interval

*p < .05, **p < .01, ***p < .001

In the adjusted model (see Model 2 in Table 5), which controls for covariates, fewer indicators in the model were associated with material hardship. However, economic self-sufficiency [B = −.61, *t* (24, 158) = − 2.70, *p* = .008], financial strain [B = .43, *t* (24, 158) = 2.11, *p* = .37] posttraumatic stress disorder [B = 1.06, *t* (24, 158) = 2.93, *p* = .004], and economic abuse [B = 1.01, *t* (24, 158) = 3.99, *p* = .000) remained statistically significant. With the exception of economic self-sufficiency, which was associated with a decrease in material hardship, the other significant indicators were associated with an increase in material hardship.

## Discussion

The purpose of this study was to explore factors associated with pregnant women’s experiences of material hardship during the COVID-19 pandemic. Analyses were guided by three research questions: (1) To what extent did pregnant women in this sample experience material hardship during the past 1 year? (2) Did women’s experiences with material hardship and associated factors differ by income level? (3) What factors were most associated with women’s experiences with material hardship in the past 1 year?

Almost three-fourths of the sample experienced at least one material hardship over the past year. On average, participants experienced two forms of material hardship. However, there were significant differences in experiences of material hardship by participants’ income level; participants at the lowest income level (i.e., less than $20,000) reported an average of four forms of material hardship. Although it is unclear how participants’ experiences of material hardship in the past 1 year differed from other years for them, approximately half the sample reported that their employment status changed during the COVID-19 pandemic as well (i.e., their hours were reduced, they lost their job, or were furloughed).

The factors significantly associated with material hardship for women in this sample were economic self-sufficiency, financial strain, posttraumatic stress disorder, and economic abuse. Economic self-sufficiency was negatively associated with material hardship, whereas financial strain, posttraumatic stress disorder, and economic abuse were all positively associated with material hardship.

Perhaps the most significant contribution that this study makes to the COVID-19 literature is the inclusion of economic abuse as part of measurement of intimate partner violence. Although researchers have highlighted the increase in intimate partner violence that has emerged as part of the COVID-19 pandemic, both in the general population [40, 42] and among pregnant women specifically [43, 44], to the author’s knowledge none of these studies included economic abuse. In this study, for every one-point increase on the SEA-2, which measured economic abuse experiences, women experienced approximately one material hardship. This is not surprising, as research suggests that the financial consequences of economic abuse experiences can be devastating to a survivors’ financial well-being [29] and that these effects can persist long after a relationship has ended [53]. Although this study did not ask women if their experiences with intimate partner violence changed as a result of the COVID-19 pandemic, these study findings are consistent with news reports that have highlighted ways in which economic abuse has been able to persist or worsen during the pandemic [54, 55].

Study findings are also consistent with research that has highlighted the association between financial stress during the COVID-19 pandemic and mental health outcomes for pregnant women [6, 8, 9]. Most of these studies found depression and/or anxiety to be significantly associated with financial strain during the pandemic; however, in this study only posttraumatic stress disorder was significantly associated with material hardship. Researchers have documented the psychological impacts of COVID-19 globally, which include posttraumatic reactions and the exacerbation of other mental health problems [56, 57]. Pregnant women also have additional added stressors associated with the pandemic, including anxiety about being infected with COVID, the effects of the vaccine on neonatal health, and concerns about whether their partner will be able to attend the delivery [10, 58]. Additional research is needed to examine whether there are significant increases in mental health challenges among pregnant women during the pandemic, compared to pre-pandemic times; this increase would not be surprising given documented increases in mental health challenges in the general population overall [56, 57].

Although other studies examining pregnant women’s mental health during the pandemic have included variables related to financial strain or something similar, none of these studies looked at other facets of financial well-being, such as financial self-sufficiency and financial self-efficacy. In this study, material hardship was significantly and positively associated with financial self-sufficiency. Typically, financial self-sufficiency refers to the ability of an individual to avoid poverty and support themselves financially free from public assistance [59]. Therefore, it is not surprising that there is an association between these two constructs, as they represent various facets of financial well-being. It is possible that participants who had lower levels of financial self-sufficiency prior to COVID-19 were more vulnerable to the pandemic’s financial impacts, thereby increasing their risk for material hardship. However, this study did not assess for changes in financial self-sufficiency as a result of the pandemic.

Several scholars have emphasized that governments will need to develop COVID-19 recovery plans that prioritize the unique needs of women [5, 33]. Stimulus packages and other financial resources may be inaccessible to the women who need them most if they continue to be distributed in ways that are not sensitive to the needs of women experiencing economic abuse and other forms of intimate partner violence. Potential ways to rectify this include allowing recipients to specify how they would like to receive their stimulus check and having clear systems in place for survivors who need to report stolen payments [55]. Although this study did not ask participants specifically if they were able to access their stimulus checks or not, economic abuse can clearly play a significant role in women’s financial insecurity.

Further, women would benefit from programs that support their financial empowerment. Within domestic violence organizations, programs commonly used to promote financial empowerment include financial literacy, career development, and individual development accounts. Other services that survivors would benefit from, particularly during times of financial crisis, include rent and utility assistance. However, not all individuals experiencing economic abuse identify themselves as victims. Therefore, it is also important for other service providers to be aware of the dynamics of economic abuse, screen for it, and be prepared to provide referrals [53]. In this study, women were not asked if they had ever received services from a domestic violence organization due to concerns their partner could be monitoring their computer. However, almost the entire sample had received prenatal services or was planning to receive them in the next month. As such, maternal health service providers offer one potential space for intimate partner violence screening that includes economic abuse to take place. These questions could be asked to patients privately in conjunction with questions about mental health more broadly, as both of these issues are associated with negative maternal and child health outcomes, including preterm birth, low birth weight, small for gestational age, and perinatal death [24–26]. Because pregnant women require frequent medical appointments, pregnancy provides “strategic moments” during which screening and referral can occur [60].

In addition, there is a need for policies and practices that increase the financial independence of women overall. The pandemic has exacerbated systemic inequalities that previously existed between men and women. Research suggests that women have incurred increased caregiving responsibilities as a result of the COVID-19 pandemic, as schools and childcare facilities closed and older adults in the family required additional support [33]. Subsequently, women have experienced losses in work productivity that may result in financial repercussions in the future, such as lost opportunities for promotion [61, 62]. Women are in need of equal pay, paid parental and sick leave, and affordable childcare options to support their participation in the work force [63].

Lastly, the United States is the only high-income country to not offer a national paid maternity leave policy [64]. Paid maternity leave is associated with a range of benefits to mothers and their children, including improved mental and physical health and reduced infant mortality [64–66]. Further, unpaid maternity leave perpetuates disparities, as low-income women may not have access to paid leave or be able to afford unpaid leave [64, 67]. As such, there is a need for maternal leave policies that increase access to paid maternity leave within the United States, as this would further support the financial well-being of women during pregnancy and post-partum.

### Limitations

This study does have some limitations that must be noted. The data collected as part of this study came from a Qualtrics survey panel. As such, this was a non-probability sample of women who self-selected to participate. The data is not generalizable and may be biased by factors such as recall or self-report bias. The data was also cross-sectional and did not ask participants about their experiences prior to COVID-19. Future research should ask women, including pregnant women, about how their financial well-being, mental health, and experiences with intimate partner violence changed as a result of the pandemic, and explore the long-lasting effects on these domains of health.

Quotas were used to ensure an income distribution across the sample, but no quotas were established for race/ethnicity; this resulted in a sample that lacked racial diversity, as the majority of participants identified as white. Future research should explore factors associated with material hardship with a more diverse sample of pregnant women. Further, to ensure participant safety, the consent form emphasized risks associated with internet privacy, particularly for individuals experiencing intimate partner violence. The response rate for this study was 41%. A total of 510 individuals accessed the survey and 457 consented to participate. However, 63 were ineligible because they were not in a romantic relationship, 137 were not currently pregnant, and the remainder did not complete the survey. It is possible that individuals experiencing the most severe intimate partner violence were unable to participate due to their own safety concerns. It is also likely that individuals underreported their abuse experiences. Although it is possible that women were more likely to complete this survey due to financial need, thereby contributing to the high rates of material hardship, the income brackets of participants were distributed relatively evenly due to quotas established prior to data collection.

Lastly, many of the measures that were used in this study (e.g., SEA2; Scale of Economic Self-Sufficiency) was developed and validated with samples of survivors who were seeking domestic violence services. Although the Cronbach’s alpha for these measures were generally between good and excellent, additional research is needed to continue to validate these measures with community samples of women.

## Conclusion

Globally, the COVID-19 pandemic has caused significant financial hardship for women and their families. Pregnant women, in particular, experience unique stressors as a result of increased financial demands associated with childbirth, concerns about access to reproductive healthcare, uncertainty about the impact of the virus on child and maternal health, and mental health vulnerabilities during the perinatal period [58]. This study explored factors associated with material hardship among a sample of pregnant women in the United States. Overall, findings from this study are consistent with others that have documented the association between financial hardship and mental health symptoms among pregnant women during the COVID-19 pandemic. A unique contribution of this study is that it highlights the significant, positive association between economic abuse, a unique form of intimate partner violence, and material hardship among pregnant women during the pandemic. These findings suggest the need for policy and practice interventions that help to ameliorate the financial insecurity experienced by some pregnant women, as well as respond to associated bidirectional vulnerabilities (e.g., mental health symptoms, experiences of intimate partner violence).

## Supplementary Information

**Additional file 1.** Checklist for Reporting results of Internet E-Surveys (CHERRIES; Eysenbach, 2004).

## Data Availability

The datasets used and/or analyzed during the current study are available from the corresponding author on reasonable request.
