# Supplementary material for: Exploring factors associated with pregnant women’s experiences of material hardship during COVID-19: a cross-sectional Qualtrics survey in the United States
Source: BMC Pregnancy Childbirth. 2021 Nov 8;21:755. doi: 10.1186/s12884-021-04234-1 (PMC8573078; doi:10.1186/s12884-021-04234-1)
Supplement: Supplementary file 1 — Additional file 1. Checklist for Reporting results of Internet E-Surveys (CHERRIES; Eysenbach, 2004). [file 12884_2021_4234_MOESM1_ESM.docx]

**Supplementary Information: Checklist for Reporting results of Internet E-Surveys (CHERRIES; Eysenbach, 2004)**

| Item Category | Checklist Item | Explanation | Description of Study |
| --- | --- | --- | --- |
| Design | Design survey design | Describe target population, sample frame. Is the sample a convenience sample? (In “open” surveys this is most likely. | The population of interest was female-identified, pregnant women living in the United States. Qualtrics panel service was used to distribute the survey to a pre-determined pool of potential respondents. As such, non-probability based sampling was used. |
| Institutional Review Board (IRB) approval and informed consent process | IRB approval | Mention whether the study has been approved by an IRB. | This study was approved by the [university] IRB. |
|  | Informed consent | Describe the informed consent process. Where were the participants told the length of time of the survey, which data were stored and where and for how long, who the investigator was, and the purpose of the study? | The survey began with informed consent, which outlined the anticipated length of time it would take to complete the survey (30 minutes), as well as the principal investigator and the purpose of the study. The informed consent also indicated that participation was voluntary and that responses were anonymous. |
|  | Data protection | If any personal information was collected or stored, describe what mechanisms were used to protect unauthorized access. | Responses were anonymous; no identifying information (including no IP addresses) were collected. |
| Development and pre-testing | Development and testing | State how the survey was developed, including whether the usability and technical functionality of the electronic questionnaire had been tested before fielding the questionnaire. | The survey was developed by the PI and included a series of validated measures. The Qualtrics program coordinator then reviewed the survey for usability and technical functionality; it was then piloted with 10% of the sample prior to fielding. |
| Recruitment process and description of the sample having access to the questionnaire | Open survey versus closed survey | An “open survey” is a survey open for each visitor of a site, while a closed survey is only open to a sample which the investigator knows (password-protected survey). | The survey was closed; only participants who received an invitation from Qualtrics panel service were able to access the survey. |
|  | Contact mode | Indicate whether or not the initial contact with the potential participants was made on the Internet. (Investigators may also send out questionnaires by mail and allow for Web-based data entry.) | Initial contact regarding the survey was made via email by Qualtrics panel service. |
|  | Advertising the survey | How/where was the survey announced or advertised? Some examples are offline media (newspapers), or online (mailing lists – If yes, which ones?) or banner ads (Where were these banner ads posted and what did they look like?). It is important to know the word of the announcement as it will heavily influence who chooses to participate. Ideally the survey announcement should be published as an appendix. | Qualtrics panel service sent a recruitment email to a pool of individuals who were potentially eligible for participation (e.g., female-identified individuals of reproductive age). |
| Survey administration | Web/E-mail | State the type of e-survey (e.g., one posted on a Web site, or one sent out through email). If it is an e-mail survey, were the responses entered manually into a database, or was there an automatic method for capturing responses? | This was a web-based survey built in Qualtrics and distributed to a pre-determined pool of potential participants through Qualtrics panel service. Responses were automatically captured. |
|  | Context | Describe the web site (for mailing list/newsgroup) in which the survey was posted. What is the Web site about, who is visiting it, what are visitors normally looking for? Discuss to what degree the content of the Web site could pre-select the sample or influence the results. | Qualtrics panel service is a sampling and online survey administration program that sends online services to targeted pool of respondents. |
|  | Mandatory/voluntary | Was it a mandatory survey to be filled in by every visitor who wanted to enter the web site, or was it a voluntary survey? | This survey was voluntary. |
|  | Incentives | Were any incentives offered (e.g., monetary, prizes, or non-monetary incentives such as an offer to provide the survey results)? | Participants were compensated approximately $10, although the exact compensation rate was designated by Qualtrics. |
|  | Time/Date | In what timeframe were the data collected? | Data were collected between January 15, 2021 and January 22, 2021. |
|  | Randomization of items or questionnaires | To prevent biases items can be randomized or alternated. | Items in the survey were not randomized. |
|  | Adaptive questioning | Use adaptive questioning (certain items, or only conditionally displayed based on responses to other items) to reduce number and complexity of the questions. | Adaptive questioning was used to reduce the number of items and complexity of items where appropriate. |
|  | Number of items | What was the number of questionnaire items per page? The number of items is an important factor for the completion rate. | The number of items per page varied depending on the nature of the questions being asked and whether they were part of a validated scale. On average, each page had 8 items. The minimum number was 1 and the maximum number was 17. |
|  | Number of screens (pages) | Over how many pages was the questionnaire distributed? The number of items is an important factor for the completion rate. | Overall, the survey questions were distributed across 20 pages. |
|  | Completeness check | It is technically possible to do consistency or completeness checks before the questionnaire is submitted. Was this done, and if “yes”, how (usually JAVAScript)? An alternative is to check for completeness after the questionnaire has been submitted (and highlight mandatory items). If this has been done, it should be reported. All items should provide a non-response option such as “not applicable” or “rather not say”, and selection of one response option should be enforced. | Given the sensitive nature of some of the survey questions, the selection of a response option was not enforced. However, incomplete surveys were excluded from the final sample. Further, participants who had missing data on the primary variables of interest were excluded from this analysis. |
|  | Review step | State whether respondents were able to review and change their answers (e.g., through a back button or a review step which displays a summary of the responses and asks respondents if they are correct). | As a safety precaution, no back button was included on the survey. However, participants did have the option of exiting the survey and completing it at a later time (within one week). |
| Response rates | Unique site visitor | If you provide view rates or participation rates, you need to define how you determined a unique visitor. There are different techniques available, based on IP addresses or cookies or both. | Cookies were used to prevent multiple entries by the same participant. |
|  | View rate (Ratio of unique survey visitors/unique site visitors) | Requires counting unique visitors to the first page of the survey, divided by number of unique site visitors (not page views!). | This information was not collected. |
|  | Participation rate (Ratio of unique visitors who agreed to participate/unique first survey page visitors) | Count the unique number of people who filled in the first survey page (or agreed to participate, for example, by checking a checkbox), divided by visitors who visit the first page of the survey (or the informed consent page, if present). This can also be called “recruitment” rate. | A total of 510 unique visitors completed the first page of the survey (i.e., informed consent). Of those, 457 consented to participate (90%). |
|  | Completion rate (Ratio of users who finished the survey/users who agreed to participate) | The number of people submitting the last questionnaire page, divided by the number of people who agreed to participate (or submitted the first survey page). This is only relevant if there is a separate “informed consent” page or if the survey goes over several pages. This is a measure for attrition. Note that “completion” can involve leaving questionnaire items blank. | Of the 457 individuals who consented to participate, 210 completed the survey (46%). |
| Preventing multiple entries from the same individual | Cookies used | Indicate whether cookies were used to assign a unique user identifier to each client computer. If so, mention the page on which the cookie was set and read, and how long the cookie was valid? Were duplicate entries avoided by preventing users access to the survey twice; or were duplicate database entries having the same user ID eliminated before analysis? In the latter case, which entries were kept for analysis (e.g., the first entry or the most recent)? | Qualtrics prevented multiple submissions by placing a cookie on the participants’ browser when they submitted a survey response. Therefore, the next time that individual clicked on the survey link, Qualtrics could see this cookie and restrict them from taking the survey again. |
|  | IP check | Indicate whether the IP address of the client computer was used to identify potential duplicate entries from the same user. If so, mention the period of time for which no two entries from the same IP address were allowed (e.g., 24 hours). Were duplicate entries avoided by preventing users with the same IP address access to the survey twice; or were duplicate database entries having the same IP address within a given period of time eliminated before analysis? If the latter, which entries were kept for analysis (e.g., the first entry or the most recent)? | To ensure the anonymity of participants, no IP addresses were collected as part of the survey. |
|  | Log file analysis | Indicate whether other techniques to analyze the log file for identification of multiple entries were used. | No other techniques were used to analyze the log file for identification of multiple entries. |
|  | Registration | In “closed” (non-open) surveys, users need to login first and it is easier to prevent duplicate entries from the same user. Describe how this was done. | This was managed by Qualtrics panel service. |
| Analysis | Handling of incomplete questionnaires | Were only completed questionnaires analyzed? Were questionnaires which terminated early (where, for example, users did not go through all questionnaire pages) also analyzed? | Only complete surveys were analyzed. |
|  | Questionnaires submitted with an atypical timestamp | Some investigators may measure the time people needed to fill in a questionnaire and exclude questionnaires that were submitted too soon. Specify the timeframe that was used as a cut-off point, and describe how this point was determined. | After the pilot, the Qualtrics program coordinator added a speeding check (measured as one-half the median soft launch time) which automatically terminated individuals who were not responding thoughtfully. The cut-off time was set at six minutes. |
|  | Statistical correction | Indicate whether any methods such as weighting of items or propensity scores have been used to adjust for the non-representative sample; if so, please describe the methods. | No statistical correction was performed. |
